# Associations of physical activity, sleep with functional disability in Chinese older adults: a five-year prospective study

**DOI:** 10.1016/j.clinsp.2026.100863

**Published:** 2026-02-12

**Authors:** Xuewei Zhao, Jiani Zeng, Lijuan Zhang, Haibo Zhang, Jingjing Huang, Lei Jiang, Jun Zhao

**Affiliations:** a Geriatric Medicine Department, The Fourth Affiliated Hospital of Nanjing Medical University, Nanjing City, Jiangsu Province, China; b School of Health Policy and Management, Nanjing Medical University, Nanjing City, Jiangsu Province, China; c School of Public Health, Nanjing Medical University, Nanjing City, Jiangsu Province, China; d School of Health Economics and Management, Nanjing University of Chinese Medicine, Nanjing City, Jiangsu Province, China; e Hospital Management Research Institution, The First Affiliated Hospital of Nanjing Medical University, Nanjing City, Jiangsu Province, China

**Keywords:** Exercise, Functional disability, Activities of daily living, Sleep, Older adults

## Abstract

**Objective:**

To explore the associations of Physical Activity (PA) and sleep with functional disability among older adults.

**Method:**

A total of 1927 participants were enrolled from the China Health and Retirement Longitudinal Study (CHARLS) between 2015 and 2020. Activities of Daily Living (ADL) were used to assess functional status. Sleep consisted of nighttime sleep (healthy sleep, or unhealthy sleep) and daytime nap sleeping (extended, moderate, short, or none), and PA was classified as sufficient or insufficient. Effects of sleep and PA on ADL Disability (ADLD) among the elderly were examined using logistic regression analysis, with Odds Ratios (ORs) and 95% Confidence Intervals (95% CIs) calculated.

**Results:**

After a follow-up of 5-years, healthy nighttime sleep was linked to a decreased odds of ADLD (OR = 0.77, 95% CI: 0.60‒0.98). In participants with sufficient PA, healthy nighttime sleep was linked to the odds of ADLD (OR = 0.68, 95% CI: 0.52‒0.90). No multiplicative interaction of PA and nighttime sleeping was found. Moreover, the association between healthy nighttime sleep and decreased risk of ADLD was consistent in females (OR = 0.64, 95% CI: 0.45‒0.91) and those with complications (OR = 0.63, 95%CI: 0.46‒0.87).

**Conclusion:**

Healthy nighttime sleep may be beneficial for preventing ADLD in older people. The benefits of sufficient PA with healthy sleep on reducing the risk of ADLD remain uncertain.

## Highlights

• Healthy nighttime sleep may be linked to lower odds of ADLD.
• Healthy sleep is linked to reduced risk of ADLD in the elderly with sufficient PA.
• Benefits of sufficient PA and healthy sleep on reducing the risk of ADLD is unclear.

## Introduction

As global aging accelerates, the number of people aged 65 and over is anticipated to reach 1.6 billion by the year 2050..^1^ In China, the number will reach 365 million, or 26.1 percent of the population.^2^ Ageing is frequently coupled with functional decline and impairment. The capacity to perform Activities of Daily Living (ADL) like bathing, dressing, cooking, and shopping is vital for older people.^3^ Despite the declined occurrence of functional dependence among the Chinese older people, the rapid aging has outweighed it, causing a large growth in the number of people needing complex care.^4^ Therefore, it is of great value to identify modifiable factors that affect the odd of Activities of Daily Living Disability (ADLD) to reduce nursing burden.

Appropriate lifestyle modification is recommended by the WHO as the “best buy” strategy to prevent Non-Communicable Diseases (NCDS).^5^ Two key lifestyle components spanning the 24-hour life cycle are sleep and Physical Activity (PA). Cross-sectional studies showed longer or shorter sleep was linked to a higher incidence of ADLD.^6^^,^^7^ A cohort study conducted by Yang^8^ suggested a link between excessive nighttime sleep and an elevated incidence of ADLD. In addition, adequate levels of PA are also considered to have a positive impact on health. Meta-analyses based mainly on European and American older adults have indicated an association of moderate/high PA levels with lower ADLD.^9^ Sleep and PA may be interdependent and work together through related pathways to affect health status.^10^ Studies have shown that low levels of PA increase mortality and type 2 diabetes in association with poor sleep.^11^^,^^12^ Beyond the independent effects on ADLD, the potential combined impacts of sleep and PA on ADLD remain unknown.

Herein, the hypothesis is that active PA may be beneficial to healthy sleep for ADLD prevention. Given the above, the verifying study intends to analyze the association of sleep and PA with ADLD, and to explore the mediating effect of PA on the risk of sleep and ADLD via the China Health and Retirement Longitudinal Study (CHARLS) data.

## Materials and methods

### Study population

This prospective cohort study used data from the CHARLS database (http://charls.pku.edu.cn/en), which examines the social, economic, and health status of community-dwelling Chinese individuals aged above 45-years. The CHARLS national baseline survey started in 2011, using a multi-stage stratified full-group sampling method to enroll > 17,000 Chinese subjects from 28 provinces. Four follow-up surveys were conducted in 2013, 2015, 2018, and 2020. In this study, the cohort from 2015 to 2020 was selected for analysis, with 2015 as the baseline. Because CHARLS belongs to public databases, the patients involved in the database have obtained ethical approval, users can download relevant data for free for research and publish relevant articles, and the present study is based on open-source data, and the Nanjing Medical University and The First Affiliated Hospital of Nanjing Medical University do not require research using publicly available data to be submitted for review to their ethics committee, so there are no ethical issues and other conflicts of interest. This study follows the STROBE Statement.

### Measurement of ADL

ADL ability assessment included basic activities of daily living (BADL; getting up, dressing, eating, toileting, bathing, continence) and instrumental activities of daily living (IADL; doing housework, shopping, cooking, administering medicine, managing finances).^13^^,^^14^ ADLD was a need for assistance or an inability to complete any of the tasks.

### Measurement of exposure

As a previous study^15^ the time range was transformed by taking the middle value, i.e.: “≥ 30-min and < 2h” was deemed as 75-min. The total PA Volume (PAV) = 8.0 × weekly vigorous PA duration + 4.0 × weekly moderate PA duration + 3.3 × weekly low-intensity PA duration. The PA was divided into sufficient PA (≥ 600 MET·min/week), and insufficient PA (< 600 MET·min/week).^16^

Sleeping between 7 and 9 h per night is deemed as healthy sleep; otherwise is unhealthy sleep.^17^ The duration of daytime naps was divided into no naps, short napping (< 30 min per day), moderate napping (30‒89 min per day), and extended napping (≥ 90 min per day).

### Potential covariates

Socio-demographic characteristics included age (years), gender (male, or female), education level (primary school and below, or junior school and above), marital status (married, or unmarried), residence (village, or town/city), and insurance (no, yes). Health-related factors included waist (cm), Body Mass Index (BMI) (kg/m^2^; < 24, or ≥ 24), grip (kg; low, or high), comorbidity, anemia (no, or yes), depression (< 10, or ≥ 10), tranquilizer pill use (no, or yes), C-Reactive Protein (CRP) (mg/L, ≤ 3, or > 3), estimated glomerular filtration rate (eGFR, < 60, or ≥ 60), smoking (current smoking, or no current smoking), drinking (current drinking, or no current drinking), and social activity (no, or yes). Supplementary Table 1 describes the definitions in detail.

### Statistical analysis

Descriptive statistics are expressed stratified by ADLD. Missing data were imputed using a multiple imputation method (Supplementary Table 2). The missing proportion of health status and annual income accounted for >50 %, the two variables were deleted. Continuous variables were tested for normality using the skewness and kurtosis methods and for homogeneity of variance using the Levene test. Normal distribution measurement data were described by mean and standard deviation [Mean(±SD)] The *t*-test was used for comparison between groups with equal variance, and the t'-test was used for unequal variance. Non-normal data were described by the median and interquartile range [M (Q₁, Q₃)], and the Wilcoxon rank sum test was used for comparison between groups. The categorical variables were described by the number of cases and the constituent ratio n ( %), and the chi-square test or Fisher's exact test was used for comparison between groups.

With ADLD as the outcome, univariate Logistic regression analysis was performed on all potential covariates, and variables with *p* < 0.05 were selected as covariates that needed to be adjusted (Supplementary Table 3). A logistic regression model analyzed the associations of sleep and PA with ADL, with Odds Ratio (OR) and 95 % Confidence Interval (95 % CI) calculated. Model 1 was an unadjusted model including only the exposure variables and outcome, and Model 2 and Model 3 were the models adjusted for covariates with *p* < 0.05 as reported..^14^^,^^18^^,^^19^ Participants were stratified according to sex and comorbidity to validate the generalizability of associations across populations. Data extraction and all subsequent analyses were performed using R version 4.2.3.

## Results

### Characteristics of participants

Initially, 9094 patients over the age of 60 were recruited, after excluding 1) No information on PA (*n* = 4895), 2) No information on nighttime sleep duration (*n* = 73), 3) No information on daytime nap duration (*n* = 25), 4) No information on ADLD (*n* = 1812), and 5) Occurrence of ADLD at baseline (*n* = 362), 1927 participants were finally included.

This prospective cohort study enrolled 1927 participants (67.86 ± 6.11-years-old, 56.88 % female), with 368 (19.10 %) individuals who developed ADLD. Table 1 summarizes the baseline characteristics of participants stratified by ADLD. Compared to the non-ADLD group, older participants, female, residing in a village, uninsured, with lower grip strength, a higher number of comorbidities, elevated depression scores, higher CRP levels, and unhealthy nighttime sleep were more likely to exhibit ADLD (*p* < 0.05).

**Table 1:** The characteristics of the included studies.

Variables	Total (*n* = 1927)	Non-ADLD (*n* = 1559)	ADLD (*n* = 368)	Statistics	p
Age, Mean (±SD)	67.86 (±6.11)	67.42 (±5.81)	69.72 (±6.95)	t' = −5.891	<0.001
Gender, n ( %)				χ² = 14.196	<0.001
Male	831 (43.12)	705 (45.22)	126 (34.24)		
Female	1096 (56.88)	854 (54.78)	242 (65.76)		
Education, n ( %)				χ² = 0.000	1.000
Primary school and below	1473 (76.44)	1192 (76.42)	281 (76.36)		
Junior school and above	454 (23.56)	367 (23.54)	87 (23.64)		
Marital status, n ( %)				χ² = 3.815	0.051
Married	1564 (81.16)	1279 (82.04)	285 (77.45)		
Unmarried	363 (18.84)	280 (17.96)	83 (22.55)		
Residence, n ( %)				χ² = 5.384	0.020
Village	1435 (74.47)	1143 (73.32)	292 (79.35)		
Town/city	492 (25.53)	416 (26.68)	76 (20.65)		
Insurance, n ( %)				χ² = 4.236	0.040
No	147 (7.63)	109 (6.99)	38 (10.33)		
Yes	1780 (92.37)	1450 (93.01)	330 (89.67)		
BMI, n ( %)				χ² = 0.000	0.996
< 24	1123 (58.28)	908 (58.24)	215 (58.42)		
≥ 24	804 (41.72)	651 (41.76)	153 (41.58)		
Waist, M (Q₁, Q₃)	85.80 (79.00, 92.50)	85.60 (79.15, 92.24)	86.50 (78.80, 92.84)	*W* = 282,377.500	0.641
Grip, n ( %)				χ² = 16.325	<0.001
Low	455 (23.61)	338 (21.68)	117 (31.79)		
High	1472 (76.39)	1221 (78.32)	251 (68.21)		
Comorbidity, Mean (±SD)	1.25 (±0.96)	1.22 (±0.96)	1.38 (±0.95)	*t* = −3.021	0.003
Anemia, n ( %)				χ² = 0.938	0.333
No	1586 (82.30)	1290 (82.75)	296 (80.43)		
Yes	341 (17.70)	269 (17.25)	72 (19.57)		
Depression, n ( %)				χ² = 15.856	<0.001
< 10	1243 (64.50)	1039 (66.65)	204 (55.43)		
≥ 10	684 (35.50)	520 (33.35)	164 (44.57)		
Tranquilizer pill use, n ( %)				‒	1.000
No	1918 (99.53)	1551 (99.49)	367 (99.73)		
Yes	9 (0.47)	8 (0.51)	1 (0.27)		
CRP, n ( %)				χ² = 4.198	0.040
≤ 3	1572 (81.58)	1286 (82.49)	286 (77.72)		
> 3	355 (18.42)	273 (17.51)	82 (22.28)		
eGFR, n ( %)				χ² = 2.311	0.128
< 60	71 (3.68)	52 (3.34)	19 (5.16)		
≥ 60	1856 (96.32)	1507 (96.66)	349 (94.84)		
Smoking, n ( %)				χ² = 0.784	0.376
Current smoking	477 (24.75)	393 (25.21)	84 (22.83)		
No current smoking	1450 (75.25)	1166 (74.79)	284 (77.17)		
Drinking, n ( %)				χ² = 12.650	<0.001
Current drinking	585 (30.36)	502 (32.20)	83 (22.55)		
No current drinking	1342 (69.64)	1057 (67.80)	285 (77.45)		
Social activity, n ( %)				χ² = 0.211	0.646
No	914 (47.43)	735 (47.15)	179 (48.64)		
Yes	1013 (52.57)	824 (52.85)	189 (51.36)		
PA, n ( %)				χ² = 2.037	0.154
Insufficient	331 (17.18)	258 (16.55)	73 (19.84)		
Sufficient	1596 (82.82)	1301 (83.45)	295 (80.16)		
Nighttime sleeping, n ( %)				χ² = 6.613	0.010
Unhealthy sleeping	1156 (59.99)	913 (58.56)	243 (66.03)		
Healthy sleeping	771 (40.01)	646 (41.44)	125 (33.97)		
Daytime nap sleeping, n ( %)				χ² = 0.463	0.927
Extended	346 (17.96)	276 (17.70)	70 (19.02)		
No	807 (41.88)	654 (41.95)	153 (41.58)		
Short	111 (5.76)	89 (5.71)	22 (5.98)		
Moderate	663 (34.41)	540 (34.64)	123 (33.42)		

SD, Standard Deviation; Q₁, 1st Quartile; Q₃, 3st Quartile; ADLD, Activities of Daily Living Disability; BMI, Body Mass Index; CRP, C-Reactive Protein; eGFR, estimated Glomerular Filtration Rate; PA, Physical Activity.

### Independent association of exposures with ADLD

**Fig. 1:**
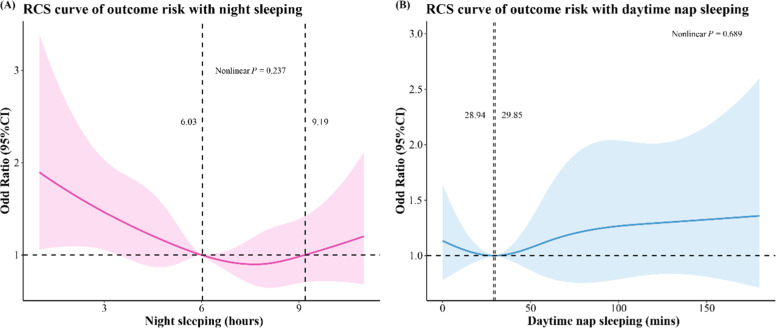
The RCS curves of nighttime sleeping, and daytime nap sleeping with ADLD. (A) Nighttime sleeping; (B) Daytime nap sleeping.

Table 2 illustrates the independent associations of PA, nighttime sleeping, and daytime nap sleeping with ADLD. The results of Model 1 suggested an association of healthy nighttime sleep with a decreased ADLD risk in the older individuals compared with unhealthy nighttime sleep (OR = 0.73, 95 % CI: 0.57‒0.92, *p* = 0.009). After adjusting for age, gender, marital status, residence, insurance, grip, comorbidity, depression, and drinking, the association remained consistent (OR = 0.77, 95 % CI: 0.60‒0.98, *p* = 0.037) in Model 2. A similar result (OR = 0.77, 95 % CI: 0.60‒0.98, *p* = 0.037) was found in Model 3 after adjustment for the above covariates and CRP. In addition, no multiplicative interaction of PA and nighttime sleeping was found (OR = 0.56, 95 % CI: 0.31‒1.04) after adjusting for age, gender, marital status, residence, insurance, grip, comorbidity, depression, CRP and drinking (Table 3). There is no obvious association of PA, daytime nap sleeping, with ADLD in either Model 2 or Model 3. Fig. 1 presents the dose-response associations of nighttime sleeping and daytime nap sleeping with ADLD. Non-linear associations of nighttime sleep (*P-*_nonlinearity_ = 0.237), daytime nap sleeping (*P-*_nonlinearity_ = 0.689) with ADLD risk were not identified.

**Table 2:** Independent associations of exposures with ADL disability.

Variables	Model 1		Model 2		Model 3	
	OR (95 % CI)	p	OR (95 % CI)	p	OR (95 % CI)	p
PA						
Insufficient	Ref		Ref		Ref	
Sufficient	0.80 (0.60‒1.08)	0.133	0.89 (0.66‒1.21)	0.466	0.90 (0.66‒1.21)	0.479
Nighttime sleeping						
Unhealthy sleeping	Ref		Ref		Ref	
Healthy sleeping	0.73 (0.57‒0.92)	0.009	0.77 (0.60‒0.98)	0.037	0.77 (0.60‒0.98)	0.037
Daytime nap sleeping						
Extended	Ref		Ref		Ref	
No	0.92 (0.67‒1.27)	0.616	0.89 (0.64‒1.24)	0.504	0.89 (0.64‒1.24)	0.499
Short	0.97 (0.56‒1.64)	0.925	0.91 (0.52‒1.60)	0.754	0.90 (0.52‒1.58)	0.734
Moderate	0.90 (0.65‒1.25)	0.520	0.92 (0.65‒1.29)	0.625	0.90 (0.64‒1.27)	0.610

ADLD, Activities of Daily Living Disability; PA, Physical Activity; OR, Odd Ratio; CI, Confidence Interval; Ref, Reference; Model 1, Adjusted for none; Model 2, Adjusted for age, gender, marital status, residence, insurance, grip, comorbidity, depression, and drinking; Model 3, Adjusted for age, gender, marital status, residence, insurance, grip, comorbidity, depression, CRP, and drinking.

**Table 3:** The multiplicative interaction of PA and nighttime on ADLD.

Variables	Model 1		Model 2	
	OR (95 % CI)	p	OR (95 % CI)	p
PA* nighttime sleeping	0.52 (0.29‒0.93)	0.028	0.56 (0.31‒1.04)	0.064

ADLD, Activities of Daily Living Disability; PA, Physical Activity; OR, Odd Ratio; CI, Confidence Interval; Ref, Reference; Model 1, Adjusted for none; Model 2, Adjusted for age, gender, marital status, residence, insurance, grip, comorbidity, depression, CRP, and drinking.

### Joint association of exposures with ADLD

Table 4 exhibits the joint association of PA, nighttime sleeping, and daytime nap sleeping with ADLD. In individuals with insufficient PA, nighttime sleeping or daytime napping was not linked to ADLD (*p* > 0.05). Whereas among the individuals with sufficient PA, healthy sleeping was linked to a lower ADLD risk in both Model 2 (OR = 0.68, 95 % CI: 0.52‒0.90, *p* = 0.007) and Model 3 (OR = 0.68, 95 % CI: 0.52‒0.90, *p* = 0.007) after adjusting for the related covariates.

**Table 4:** Joint associations of exposures with ADLD.

Variables	Model 1		Model 2		Model 3	
	OR (95 % CI)	p	OR (95 % CI)	p	OR (95 % CI)	p
**Insufficient PA**						
Nighttime sleeping						
Unhealthy sleeping	Ref		Ref		Ref	
Healthy sleeping	1.23 (0.73‒2.08)	0.435	1.20 (0.68‒2.12)	0.531	1.21 (0.68‒2.15)	0.508
Daytime nap sleeping						
Extended	Ref		Ref		Ref	
No	1.27 (0.62‒2.75)	0.520	1.34 (0.60‒3.01)	0.473	1.33 (0.59‒3.00)	0.487
Short	2.25 (0.61‒7.65)	0.201	3.17 (0.78‒12.96)	0.108	3.17 (0.77‒13.05)	0.110
Moderate	1.33 (0.62‒2.97)	0.466	1.56 (0.67‒3.63)	0.306	1.54 (0.66‒3.61)	0.318
**Sufficient PA**						
Nighttime sleeping						
Unhealthy sleeping	Ref		Ref		Ref	
Healthy sleeping	0.64 (0.48‒0.83)	0.001	0.68 (0.52‒0.90)	0.007	0.68 (0.52‒0.90)	0.007
Daytime nap sleeping						
Extended	Ref		Ref		Ref	
No	0.86 (0.61‒1.22)	0.384	0.81 (0.57‒1.17)	0.262	0.81 (0.57‒1.17)	0.259
Short	0.82 (0.44‒1.47)	0.525	0.73 (0.39‒1.36)	0.320	0.73 (0.39‒1.35)	0.314
Moderate	0.83 (0.58‒1.19)	0.299	0.83 (0.57‒1.20)	0.320	0.83 (0.56‒1.20)	0.314

ADLD, Activities of Daily Living Disability; PA, Physical Activity; OR, Odd Ratio; CI, Confidence Interval; Ref, Reference. Model 1, Adjusted for none. Model 2: Adjusted for age, gender, marital status, residence, insurance, grip, comorbidity, depression, and drinking. Model 3: Adjusted for age, gender, marital status, residence, insurance, grip, comorbidity, depression, CRP, and drinking.

### The association of exposures with ADLD in different subgroups

**Fig. 2:**
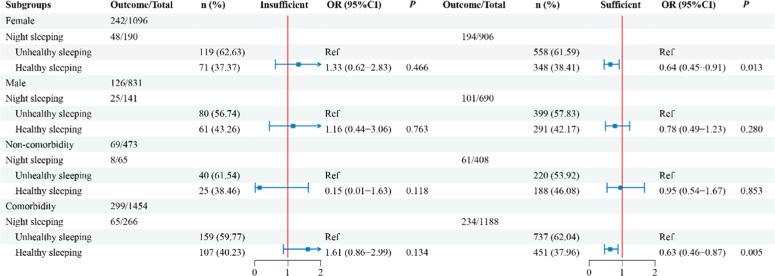
The association of exposures with ADLD in different subgroups.

Stratified participants by gender and comorbidity, the results are presented in Fig. 2. A decreased risk of ADLD with healthy nighttime sleeping, as compared to unhealthy nighttime sleep, was observed in the female (OR = 0.64, 95 % CI: 0.45‒0.91, *p* = 0.013) and with comorbidity (OR = 0.63, 95 % CI: 0.46‒0.87, *p* = 0.005) subgroups.

### Discussion

In this study, healthy nighttime sleep was linked to reduced ADLD risk in older subjects, especially in the elderly who had adequate PA. The association was also observed in females and those with comorbidities. No interaction of PA and nighttime sleeping was detected.

The study’s results are in line with other earlier studies. Wang et al. found that sleep of 8 and 10 h was linked to a lower ADLD risk, while sleep ≥12 h was linked to a higher ADLD risk in the oldest individuals.^7^ A cohort study focusing on centenarians indicated that those who slept >9 h and experienced worse quality of sleep faced an elevated hazard of moderate or severe ADLD than individuals maintaining sleep of 7 to 9 h with good sleep quality.^8^ Peng et al.,^20^ conducted a prospective study, recruiting 8361 participants aged 45 and older not affected by IADL disability in 2011, and followed them until 2018, finding that insufficient and prolonged nighttime sleep were linked to an elevated IADL disability risk in adults of middle age and older. A study on older adults from a Brazilian community found that older individuals with sleep disturbances had increased disability on the BADL and IADL compared with individuals without sleep disturbances.^21^ This association can be explained by a number of physiological mechanisms, including: 1) The key functions of sleep are reorganization, repair, maintenance of cerebral health, and waste debris.^22^^,^^23^ It has been found that sleep-restricted animals show greater Aβ plaque deposition,^24^ which plays an important role in inducing neurodegeneration.^25^^,^^26^ Neurodegeneration impairs the regeneration of muscle fibers and reduces the ability to generate muscle force,^27^ which is directly related to dysfunction. In addition, during sleep, some enzymes can repair damage to brain cells by free radicals. Due to the inability of neurotransmitters and neurons to rest or regenerate,^28^ sleep deprivation prevents our brain from functioning properly. 2) Poor sleep quality can lead to mental fatigue, which can lead to physical disability, impairing older people’s ADL function.^29^ 3) Executive functions in the prefrontal cortex, acting in concert with the anterior cingulate and posterior parietal systems, appear to be particularly vulnerable to sleep deprivation.^30^ The decline of executive attention and working memory can affect the performance of ADL in the elderly. 4) Sleep affects the levels of hormones, including growth hormone and cortisol, which are responsible for the physiological repair, nerve cell growth, and muscle repair,^31^^,^^32^ and then affects ADL function. In conclusion, the authors suggest that improved sleep quality may help maintain physical performance and independence among the older, which provides a promising intervention for lifestyle.

Beyond sleep, active exercise has also been suggested to improve ADLD in older adults. The prospective study enrolled 211 older subjects aged above 60-years and those who were sufficiently active had lower odds of disability, particularly in the areas of occupational and leisure time exercise.^33^ A community-based meta-analysis of older adults reported that moderate/high PA levels conferred a reduced risk of incident ADLD compared with low PA.^9^ Being active can also help improve the quality of your sleep. A meta-analysis shows the most positive improvements in various sleep outcomes of exercise programs among generally healthy older adults.^34^ This may be due to the fact that PA helps to improve sleep quality,^34^ while also increasing muscle strength and endurance.^35^^,^^36^ In the present study, an independent relationship was not observed between PA and ADLD among the elderly. In the further combined effect, the authors observed that adequate PA combined with healthy sleep was not associated with lower odds of ADLD in the elderly. No multiplicative interaction of PA and nighttime sleeping was found on ADLD. However, among old adults who had sufficient PA, the authors found that healthy sleeping was linked to lower odds of ADLD. The differences in results may be related to measurement error in physical activity, potential unmeasured confounding, and limited sample size.

Subgroup analysis revealed a more robust relationship of sleep quality with ADLD in the female elderly group. This may be due to the higher demand for sleep quality in females, which may exacerbate sleep disorders in ADLD. Overall, females tend to sleep more than males at most stages of the life course, possibly due to sociological findings indicating that females engage in more unsalaried labor and have less free-time quality than males.^37^ A longitudinal study of aging from Taiwan reported that older females who habitually exercised had better ADL function and retention than older men.^38^ In addition, the present results also found that comorbidities may increase the sensitivity of the elderly to health interventions, which may be related to factors such as sleep and adequate PA, reducing inflammatory response and improving physiological function and psychological state in this group.^39^^,^^40^ Future research can further explore the specific content of comorbidities. In general, this study suggests that PA and adequate sleep may be more beneficial in females and those with comorbidities and that differences in sex and health status should be considered when designing interventions.

Despite the important findings provided in this study, several limitations remain. First of all, CHARLS sampled half of the survey population to investigate PA, but it could not represent the overall sample because there was no clear sample weight. In the present study, although the sensitivity analysis (e.g., multiple imputation) was performed, the two variables (health status and annual income) were deleted due to over 50 % of data missing. Only 1927 old adults were included, which may have a selection bias. Whether the results are consistent in populations with missing data remains to be determined. More well-designed studies with complete data are performed to validate the present findings. Second, the study classifies sleep and physical activity based on self-report, which may introduce information bias due to participant subjectivity. Future studies should consider using objective measures, such as accelerometry and polysomnography, to improve the accuracy of the data. Third, although the study adjusted for several covariates (age, gender, comorbidities, among others), there are potential unmeasured factors that should be considered in future studies, such as diet, medication use, sleep quality, fragmentation, or disorders (e.g., sleep apnea) affecting sleep, or more detailed socioeconomic status. Finally, further intervention researches are warranted to elucidate the synergistic protective benefits of active PA and healthy sleep on ADLD in the elderly.

## Conclusion

Healthy nighttime sleep may be linked to a reduced ADLD risk, especially in females as well as those with comorbidities. Although the association of sufficient PA with ADLD was not found, healthy nighttime sleep was also associated with a decreased risk of ADLD in the elderly with sufficient PA. The benefits of sufficient PA with healthy sleep on reducing the risk of ADLD remain uncertain. Future studies need to further validate whether sufficient PA may play a positive role in healthy sleep on ADLD risk.

## Abbreviations

PA, Physical Activity; FD, Functional Disability; CHARLS, China Health and Retirement Longitudinal Study; ADL, Activities of Daily Living; ADLD, Activities of Daily Living Disability; ORs, Odd Ratios; CI, Confidence Intervals; NCDS, Non-Communicable Diseases; PAV, Physical Activity Volume; CRP, C-Reactive Protein.

## Ethics approval and consent to participate

The institutional review board of Peking University approved the conduct of CHARLS (Approval n° IRB00001052–11,015). All participants provided written informed consent prior to participation (https://charls.charlsdata.com/index/zh-cn.html). Because CHARLS belongs to public databases, the patients involved in the database have obtained ethical approval, users can download relevant data for free for research and publish relevant articles, and the present study is based on open-source data, and the Nanjing Medical University and The First Affiliated Hospital of Nanjing Medical University do not require research using publicly available data to be submitted for review to their ethics committee, so there are no ethical issues and other conflicts of interest.

## Consent for publication

Not applicable, because this paper did not reveal any personal information of patients.

## Data availability

The datasets used and/or analyzed during the current study were publicly available from the CHARLS database. All data used and/or analyzed are also available from the corresponding author on reasonable request.

## Authors’ contributions

(1) Xuewei Zhao and Jun Zhao conceiving and designing the study; (2) Xuewei Zhao, Jiani Zeng, Haibo Zhang, Lei Jiang collecting the data; (3) Jiani Zeng and Lei Jiang analyzing and interpreting the data; (4) Xuewei Zhao writing the manuscript; (5) Jingjing Huang, Lijuan Zhang and Jun Zhao providing critical revisions that are important for the intellectual content; (6) All authors approving the final version of the manuscript.

## Conflicts of interest

The authors declare no conflicts of interest.

## Appendix Supplementary materials

Image, application 1
