# Supplementary material for: Associations of physical activity, sleep with functional disability in Chinese older adults: a five-year prospective study
Source: Clinics (Sao Paulo). 2026 Feb 12;81:100863. doi: 10.1016/j.clinsp.2026.100863 (PMC12915216; doi:10.1016/j.clinsp.2026.100863)
Supplement: Supplementary file 1 [file mmc1.docx]

**CLINICS-D-25-00159_Supplemnetary materials**

**Supplementary Table 1** The definition of the covariates.

| **Covariates** | **Definition or CHARLS code of covariates** |
| --- | --- |
| Comorbidities of chronic diseases | According to the number of 14 chronic diseases answered in DA007. Diabetes, hypertension, dyslipidemia, and kidney disease were judged by both laboratory tests and self-report. Laboratory diagnosis of diabetes was defined as fasting blood glucose ≥7.0 mmoL/L or HbAlc≥6.5% or self-reported diabetes. Hypertension was defined as self-reported hypertension or systolic blood pressure ≥130 mmHg or diastolic blood pressure ≥80 mmHg. Dyslipidemia was defined as TC ≥200 mg/dL (5.2 mmoL/L) or TG ≥150 mg/dL (1.7 mmoL/L) or LDL-C ≥ 130 mg/dL (3.4 mmoL/L) or HDL-C ≤40 mg/dL (1.0 mmoL/L) or self-reported dyslipidemia. Nephropathy was defined by self-report or eGFRCKD-EPI < 60 mL/ min/1.73 m^2^ |
| eGFR_CKD-EPI_ | eGFRCKD-EPI = 141×min (Scr/κ, 1) ^α^ ×max(Scr/κ, 1)-1.029 ×0.993 age ×1.108 (if female) ×1.159 (if black). κ is 0.7 for females and 0.9 for males, α is -0.329 for females and -0.411 for males, min indicates the minimum of Scr/κ or 1, and max indicates the maximum of Scr/κ or 1. Scr is measured in mg/dL |
| Health insurance | Health insurance was defined according to CARD20 |
| Smoking status | Smoking status was determined according to DA059 and DA061 |
| Drinking status | Drinking status was determined according to DA067and DA069 |
| Depression | Depression was assessed using the CES-D 10. DC009-DC018, each response is scored on a scale of 0 to 3 (total scores range from 0 to 30), with a score of 10 or higher indicating depressive symptoms |
| Self-rated health status | Self-rated health status was assessed using the DA079 |
| Social activities | According to the DA056 assessment, those who choose any activity are considered to have social activities |
| Anemia | Anemia was defined as an Hb level < 13 g/dL in male and < 12 g/dL in female |
| BMI | BMI ≥24 kg/m^2^ is considered overweight or obese |
| Grip | The maximum value of grip strength measured can be classified according to the AWGS2019 standard: male grip strength < 28 kg and female grip strength < 18 kg are defined as low grip strength |

CHARLS, China Health and Retirement Longitudinal Study, HbAlc, Glycated Hemoglobin; TC, Total Cholesterol; TG, Triglycerides; LDL, Low-Density Lipoprotein; HDL, High-Density Lipoprotein; eGFR, Estimated Glomerular Filtration Rate; Scr, Serum Creatinine; CES-D, Center for Epidemiologic Studies Depression Scale; Hb, Hemoglobin; BMI, Body Mass Index.

**Supplementary Table 2** Missing data.

| **Variable** | **n** | **Proportion** | **Method** |
| --- | --- | --- | --- |
| Smoking | 3 | 0.16% | Multiple imputation |
| Residence | 27 | 1.4% | Multiple imputation |
| Depression | 188 | 9.76% | Multiple imputation |
| Weight | 252 | 13.08% | Multiple imputation |
| Waist | 252 | 13.08% | Multiple imputation |
| Height | 255 | 13.23% | Multiple imputation |
| Education | 259 | 13.44% | Multiple imputation |
| Grip | 268 | 13.91% | Multiple imputation |
| eGFR | 498 | 25.84% | Multiple imputation |
| CRP | 499 | 25.9% | Multiple imputation |
| Anemia | 504 | 26.15% | Multiple imputation |
| Health status | 1001 | 51.95% | Delete |
| Annual income | 1427 | 74.05% | Delete |

eGFR, Estimated Glomerular Filtration Rate; CRP, C-Reactive Protein.

**Supplementary Table 3** The associations between potential covariates with ADLD.

| **Variables** | **OR (95% CI)** | **p** |
| --- | --- | --- |
| Age | 1.06 (1.04‒1.08) | <0.001 |
| Gender |  |  |
| Male | Ref |  |
| Female | 1.59 (1.25‒2.01) | <0.001 |
| Education |  |  |
| Primary school and below | Ref |  |
| Junior school and above | 1.01 (0.77‒1.31) | 0.967 |
| Marital status |  |  |
| Married | Ref |  |
| Unmarried | 1.33 (1.01‒1.75) | 0.043 |
| Residence |  |  |
| Village | Ref |  |
| Town/city | 0.72 (0.54‒0.94) | 0.017 |
| Insurance |  |  |
| No | Ref |  |
| Yes | 0.65 (0.44‒0.96) | 0.031 |
| BMI |  |  |
| < 24 | Ref |  |
| ≥ 24 | 0.99 (0.79‒1.25) | 0.949 |
| Waist | 1.00 (0.99‒1.00) | 0.700 |
| Grip |  |  |
| Low | Ref |  |
| High | 0.59 (0.46‒0.76) | <0.001 |
| Comorbidity | 1.20 (1.06‒1.35) | 0.003 |
| Anemia |  |  |
| No | Ref |  |
| Yes | 1.17 (0.87‒1.56) | 0.297 |
| Depression |  |  |
| < 10 | Ref |  |
| ≥ 10 | 1.61 (1.28‒2.02) | <0.001 |
| Tranquilizer pill use |  |  |
| No | Ref |  |
| Yes | 0.53 (0.07‒4.24) | 0.548 |
| CRP |  |  |
| ≤ 3 | Ref |  |
| > 3 | 1.35 (1.02‒1.78) | 0.034 |
| Smoking |  |  |
| Current smoking | Ref |  |
| No current smoking | 1.14 (0.87‒1.49) | 0.341 |
| Drinking |  |  |
| Current drinking | Ref |  |
| No current drinking | 1.63 (1.25‒2.13) | <0.001 |
| Social activity |  |  |
| No | Ref |  |
| Yes | 0.94 (0.75‒1.18) | 0.605 |

ADLD, Activities of Daily Living Disability; OR, Odd Ratio; CI, Confidence Interval; Ref, Reference; BMI, Body Mass Index; CRP, C-Reactive Protein.
